# IgG4-related hypertrophic pachymeningitis with cerebral venous thrombosis

**DOI:** 10.1055/s-0043-1772600

**Published:** 2024-01-25

**Authors:** Arthur da Veiga Kalil Coelho, Alexia Carneiro de Almeida, Sonia Maria Cesar de Azevedo Silva Moura Magalhães Gomes, Antonio José da Rocha, Herval Ribeiro Soares Neto

**Affiliations:** 1 Hospital do Servidor Público Estadual de São Paulo, Departamento de Neurologia, São Paulo SP, Brazil.; 2 Universidade Federal de São Paulo, Escola Paulista de Medicina, Departamento de Neurologia, São Paulo SP, Brazil.; 3 Santa Casa de São Paulo, Faculdade de Ciências Médicas, Departamento de Radiologia, São Paulo SP, Brazil.; 4 Hospital Israelita Albert Einstein, Departamento de Neurologia, São Paulo SP, Brazil.

**Figure 1:**
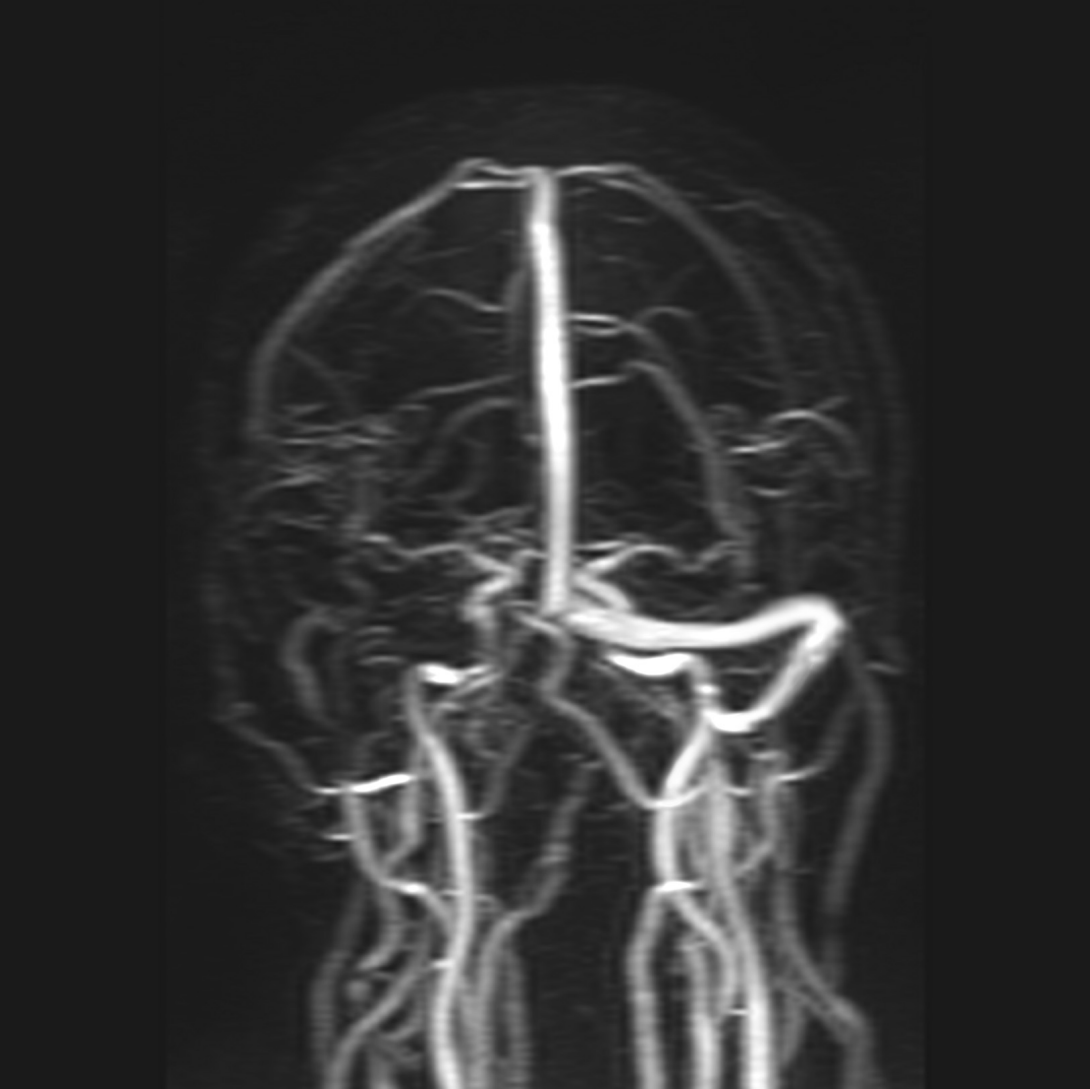
Venous magnetic resonance angiography showing venous thrombosis at the right side.

**Figure 2:**
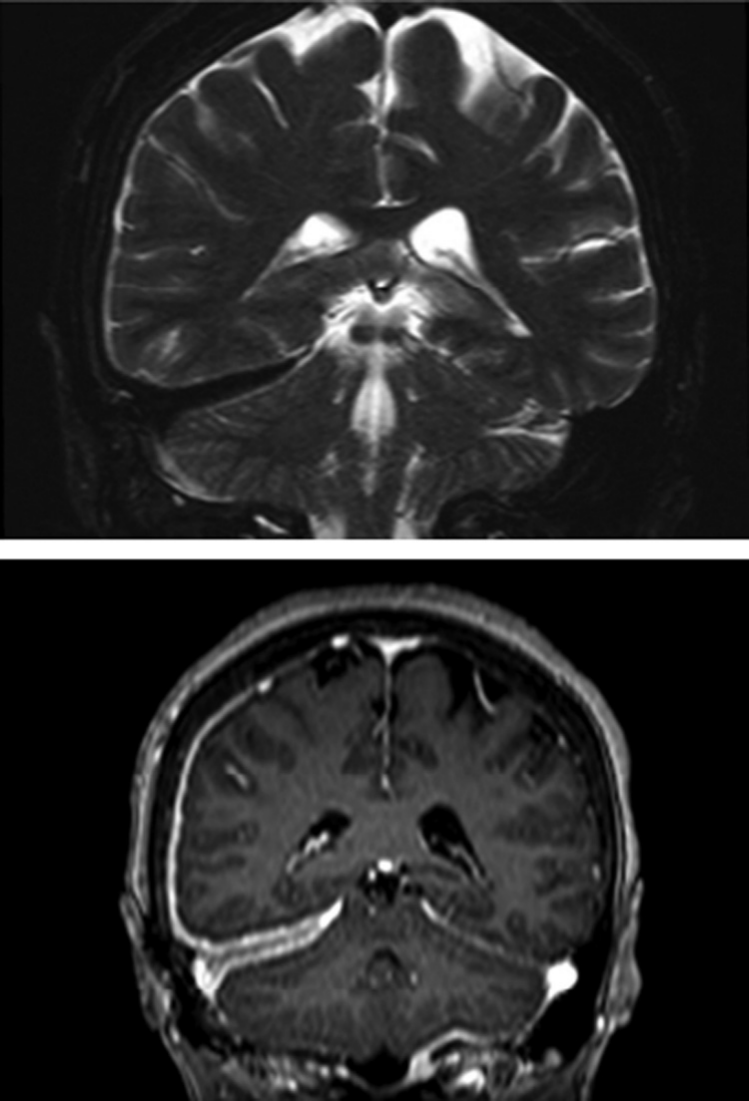
T2 (upper) and postcontrast T1 (bottom) weighted magnetic resonance imaging (MRI) scans, showing dural thickening on coronal view before treatment.

**Figure 3:**
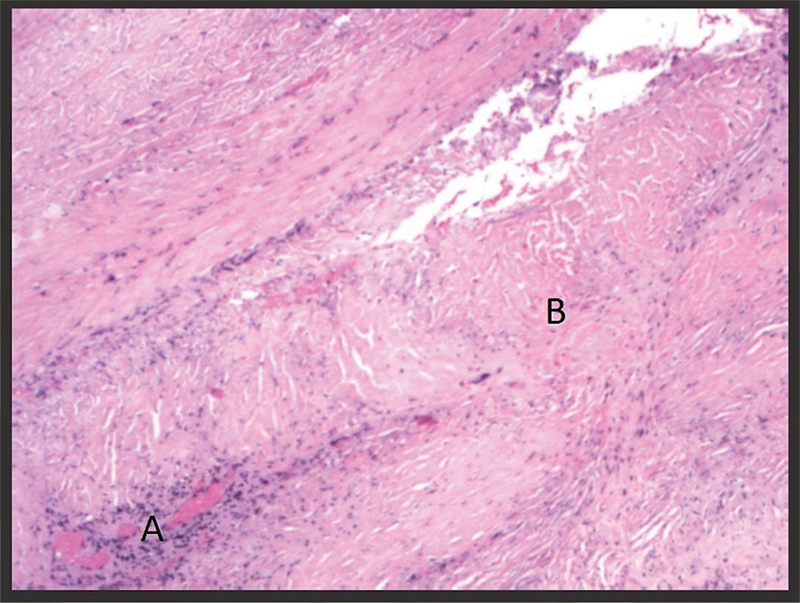
Meningeal biopsy in hematoxylin and eosin stain demonstrating dense lymphoplasmacytic infiltrate ( **A** ) and storiform fibrosis ( **B** ), two of the three histopathological criteria of IgG4-related disease.

**Figure 4:**
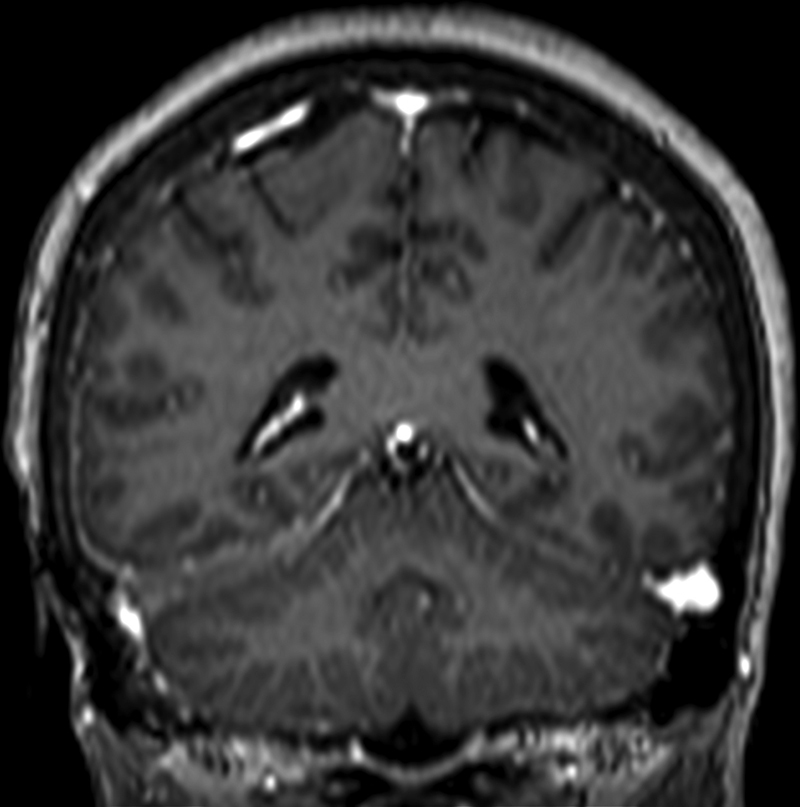
Postcontrast weighted MRI scan showing improvement in dural thickening at the right side on coronal view after treatment with rituximab.

A 58-year-old female patient presented with a 4-year history of right-sided headache with tinnitus. She had been hospitalized previously due to dysfunction of multiple cranial nerves, such as diplopia, right hemiface hypoesthesia, right peripheral facial palsy, right hearing loss, dysphonia, and dysphagia, followed by venous thrombosis of the right sigmoid sinus ( Figure 1 ), which was treated with warfarin. The cerebrospinal fluid yielded 90 leukocytes/mm ^3^ (92% of lymphocytes) and 158 mg/dL of proteins. A magnetic resonance imaging (MRI) scan revealed tentorium-temporo-parietal hypertrophic pachymeningitis ( Figure 2 ). A meningeal biopsy demonstrated dense lymphoplasmacytic infiltrate and storiform fibrosis ( Figure 3 ), two of the three histopathological criteria. 1 2 Therefore, the diagnosis was made, and rituximab 3 4 was initiated, with a very important response in terms of symptoms and imaging exams ( Figure 4 ).
